# Trafficking mechanisms underlying Na_v_ channel subcellular localization in neurons

**DOI:** 10.1080/19336950.2019.1700082

**Published:** 2019-12-14

**Authors:** Laura Solé, Michael M. Tamkun

**Affiliations:** a Molecular, Cellular and Integrative Neurosciences Graduate Program, Colorado State University, Fort Collins, CO, USA; b Department of Biomedical Sciences, Colorado State University, Fort Collins, CO, USA; c Department of Biochemistry and Molecular Biology, Colorado State University, Fort Collins, CO, USA

**Keywords:** Voltage-gated sodium channel, Nav1.6, trafficking, axon initial segment, plasticity, ion channel localization, channelopathies

## Abstract

Voltage gated sodium channels (Na_v_) play a crucial role in action potential initiation and propagation. Although the discovery of Na_v_ channels dates back more than 65 years, and great advances in understanding their localization, biophysical properties, and links to disease have been made, there are still many questions to be answered regarding the cellular and molecular mechanisms involved in Na_v_ channel trafficking, localization and regulation. This review summarizes the different trafficking mechanisms underlying the polarized Na_v_ channel localization in neurons, with an emphasis on the axon initial segment (AIS), as well as discussing the latest advances regarding how neurons regulate their excitability by modifying AIS length and location. The importance of Na_v_ channel localization is emphasized by the relationship between mutations, impaired trafficking and disease. While this review focuses on Na_v_1.6, other Na_v_ isoforms are also discussed.

## Introduction

Voltage-gated sodium channels (Na_v_) are essential for the initiation and propagation of most action potentials (APs). There are 9 isoforms of the pore-forming α subunits (Na_v_1.1–9). Na_v_1.1, Na_v_1.2, Na_v_1.3 and Na_v_1.6 are primarily expressed in the central nervous system (CNS), while Na_v_1.7, Na_v_1.8 and Na_v_1.9 are predominant in the peripheral nervous system. Na_v_1.4 and Na_v_1.5 are mostly expressed in skeletal muscle and in heart, respectively [1,2]. Each functional Na_v_ is formed by a multimer of a α subunit with 4 homologous domains (each with 6 transmembrane domains) and at least one auxiliary ß subunit. There are 4 different ß subunits (ß1-4), all of them being single-span transmembrane proteins with an extracellular N-terminus. While ß1 and ß3 associate non-covalently with the α subunit through their N- and C-terminal domains, ß2 and ß4 associate through a disulfide link with a cysteine within their N-terminal domain. Association with ß subunits can affect Na_v_ channel function, location and trafficking [3].

**Figure 1.**
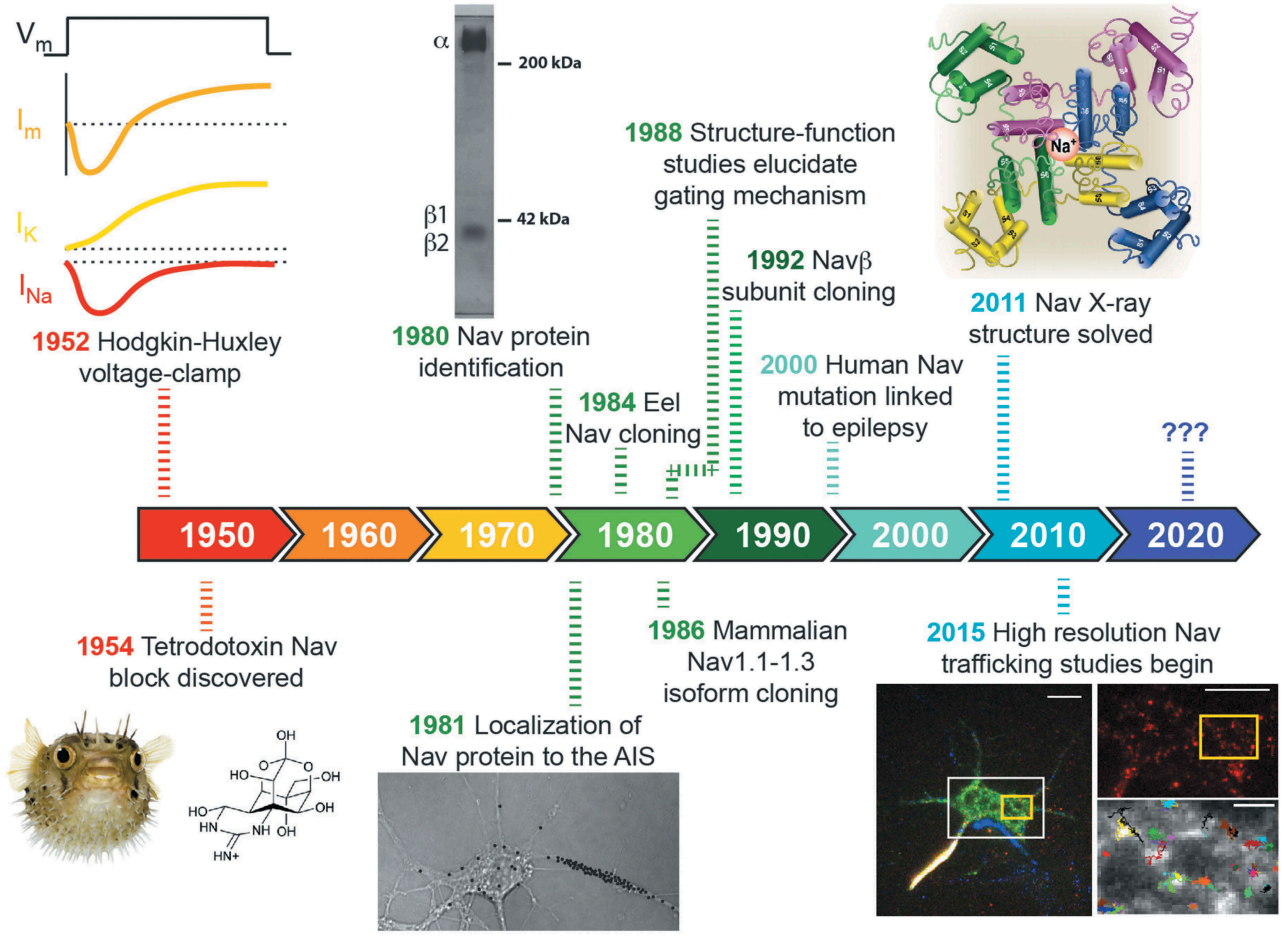
**Seventy years of Na_v_ channel history: From the squid giant axon to single molecule tracking**. The pufferfish, TTX and crystal structure images are used with permission from ref. 2 (https://doi.10.1021/jm501981g; Further permissions related to the excerpted material should be directed to the American Chemical Society). The SDS gel, autoradiography, and single molecule tracking images have not been published previously but relate to refs. 9, 11, and 56, respectively.

The first identification of protein representing a Na_v_ channel involved the purification of tetrodotoxin (TTX) binding activity from the electric eel [4], which occurred almost 30 years after the classic Hodgkin and Huxley studies [5–7], as illustrated by the time line in Figure 1. This work was soon followed by studies done in the Catterall lab characterizing the rat brain Na_v_ protein [8–10] and establishing the concentration of Na_v_ channels at the axon initial segment (AIS) [11]. The cloning of the first Na_v_ channel from the electric eel by the Numa group [12] was followed quite rapidly by the identification of the first three mammalian Na_v_ isoforms [13,14]. Numerous structure function studies then dissected the gating and ion permeation mechanisms [15–21] and specific Na_v_ mutations were linked to human disease [22–24]. Progress in high resolution structure determination [25–27] added a physical context to much of the site-directed mutagenesis work that had occurred over the previous 20 years. Recent progress in Na_v_ channel biology focuses on the mechanisms underlying channel trafficking and localization and how localization determines function [28,29], for proper nerve function requires the precise targeting of Na_v_ isoforms to distinct subcellular domains. While this review will mainly focus on the localization and trafficking of Na_v_1.6, other Na_v_ channel isoforms will be discussed when appropriate.

### Discovery of Na_v_1.6

Na_v_1.6, encoded by the SCN8A gene, had several names during the early years of cloning, such as Rat6, NaCh6, Scn8a, PN4 and CerIII. Na_v_1.6 was first described almost simultaneously by two different labs in 1995, from two different species. Schaller et al. were the first to report the full sequence of the rat Na_v_1.6, and detected its expression in brain by RT-PCR [30]. These investigators were surprised that, while Na_v_1.6 mRNA was one of the most abundant Na_v_ channel transcripts in the nervous system, it had not been cloned earlier (the mammalian Na_v_1.1–1.3 channels were first cloned in the mid-1980s [12,31]). They also demonstrated that it was expressed both in neurons and glia, with a broad distribution in CNS that was distinct from previously reported Na_v_ channels. For example, they found Na_v_1.6 in hippocampal pyramidal and granule cells, motor neurons of the spinal cord and brain stem, axons in the retina, and dendrites of cerebral cortical pyramidal cells. In parallel, Burgess et al., isolated a mouse Na_v_ channel gene, Scn8a, which was highly expressed in brain and spinal cord but not in skeletal muscle or heart, and encoded a predicted protein of 1,732 amino acids [32]. Interestingly, Scn8a was located in the flanking region of a transgene-induced allele of *med* (a mouse neurological mutant with **m**otor **e**ndplate **d**isease). This phenotype is characterized by early onset progressive paralysis of the hind limbs, severe muscle atrophy, degeneration of Purkinje cells and juvenile lethality. Later, Vega-Saenz et al., in 1997, identified expression of rat Na_v_1.6 by single-cell RT-PCR in cerebellum and cerebellar Purkinje cells [33]. In agreement with Schaller et al., rat Na_v_1.6 mRNA, as detected by in situ hybridization, was found in many neuronal populations of the CNS, being a channel highly expressed in the brain and spinal cord. Furthermore, Na_v_1.6 was proposed to be responsible for the persistent and resurgent currents observed in Purkinje cells, since these currents were decreased in cultures of Scn8a null mice [34]. Na_v_1.6 was also characterized as one of the major contributors to sodium current in mouse postnatal motor neurons [35]. The first functional characterization of mouse Na_v_1.6 was performed by Smith et al. in *Xenopus* oocytes, where they observed that Na_v_1.6 currents inactivated faster than other Na_v_ channel isoforms and presented a unique persistent current, which they concluded was responsible for distinct sodium conductances necessary for the repetitive firing of AP in Purkinje neurons [36]. Na_v_1.6 protein was first described as concentrating at Nodes of Ranvier [37–39], but was later detected in axon initial segments of Purkinje neurons [40] and cerebellar granule cells [41], and in dendrites of pyramidal cells of the cortex, hippocampus and cerebellum [42]. Na_v_1.6 was also expressed at lower levels in the somato-dendritic compartment [43,44] where it is likely responsible for the generation of dendritic action potentials [45].

### Na_v_1.6 and disease

Mutations in mouse Na_v_1.6 have been associated with ataxia, muscle weakness, tremor, dystonia and juvenile lethality [32,46,47], and conditional deletion of Scn8a in mouse cerebellar neurons resulted in mild ataxia [48]. However, it was not until the mid-2000s that the first human mutation in SCN8A was found in a patient with mental retardation, ataxia and cerebellar atrophy [49]. Later, in 2012, mutations in SCN8A were associated for the first time with early infantile epileptic encephalopathy (EIEE13, OMIM #614558). The first epileptic-related SNC8A *de novo* mutation described was in a 15-year-old female who had severe epileptic encephalopathy, some features of autism, intellectual disability, ataxia and died of SUDEP (**s**udden **u**nexplained **d**eath in **ep**ilepsy) [50]. Subsequently, and thanks primarily to advances in genome sequencing technology, the number of clinical cases associated with Na_v_1.6 has increased significantly, with more than 140 patients now diagnosed with SCN8A mutations [51]. Mutations in SCN8A are also associated with benign familial infantile seizures-5 (BFIS5, OMIM #617080) [52–54]. In a meta analysis of epilepsy genetics research over the past decade, from 5185 papers published from 2009 to 2018, 4% of the “occurrences” related with epilepsy corresponded to SCN8A mutations [55].

### Subcellular localization of Na_v_1.6

As mentioned earlier, Na_v_1.6 is highly concentrated at the AIS and Nodes of Ranvier, where it plays a major role in AP generation and propagation, respectively. The high density of Na_v_ channels in the AIS versus soma has been under debate for several decades, mainly due to disagreements between results obtained using different experimental approaches. However, the more recent publications conclude that there are 30–50 times more Na_v_1.6 channels at the AIS than in the somatic compartment [43,56,57], which contributes to the lower threshold required for AP generation in this compartment [58]. The AIS is 10–60 µm in length and characterized by a concentration of Na_v_ and K_v_ channels, cell adhesion molecules (L1 CAM, NrCAM, NF186, etc), cytosolic scaffolding proteins (AnkyrinG), a highly organized cytoskeleton (actin rings and microtubule fascicles), and microtubule-associated proteins such as TRIM46 (see reviews [59,60] for more details). Briefly, AnkyrinG (AnkG), a submembranous scaffolding protein with 270 and 480 KDa isoforms, is the major structural orchestrator of the AIS [61,62]. On its C-terminal side, it binds to microtubules through association with microtubule-associated proteins such as EB1/3 and Ndel1 [63,64]. On its N-terminal side, AnkG binds to αII- and ßIV- spectrins [62,65], which in turn associate with actin [66]. STORM super-resolution experiments demonstrated that actin, spectrin and AnkG form a periodic structure with actin filaments organized in rings spaced exactly 190 nm apart, which corresponds to the length of spectrin tetramers, and AnkG is located at the center of the spectrin tetramer [67,68]. AnkG also anchors Na_v_ and K_v_7 channels and cell adhesion molecules, which again present a 190 nm striped pattern [67–69]. While Na_v_ channels associate with AnkG through a 9 aa motif located in the intracellular II-III loop of Na_v_ channels (Ankyrin Binding Motif (ABM)) [70,71], K_v_7.2 and K_v_7.3 bind to AnkG through a Na_v_-homologous motif located in their C-terminal domain [72]. Interestingly, K_v_1 channels are also concentrated at the AIS [73] and present a 190 nm striped pattern [74], but do not bind to AnkG, and instead they cluster at the AIS by interacting with the postsynaptic density-93 (PSD-93) scaffolding protein [75]. In some cells, K_v_2 channels and Ca_v_ channels are also enriched at the AIS [76,77].

Na_v_ channel concentration at the AIS via interaction with AnkG was demonstrated independently by two labs at almost the same time [70,71]. A motif located in the intracellular II-III loop of Na_v_ channels was found to be sufficient for concentrating chimeric proteins (CD4, NF186 and K_v_2.1) to the AIS via AnkG binding. Garrido et al. demonstrated that 27 aa located at the Na_v_1.2 II-III loop (amino acids 1102–1028) governed compartmentalization of the CD4-Na_v_1.2 chimera at the AIS. Furthermore, over-expression of the soluble Na_v_1.2 II-III loop tagged with GFP decreased by half the sodium currents recorded in neurons, and dramatically decreased Na_v_ localization at the AIS. Lemaillet et al. refined the ABM present in the II-III loop of Na_v_ channels to just 9 aa (aa 1105–1113 for rNa_v_1.2) using both Na_v_1.2-GST pull-down experiments and chimera expression in hippocampal neuron cultures. Importantly, this 9 aa motif is conserved across all vertebrate Na_v_ channels ((V/A)P(I/L)AXXE(S/D)D). Both studies indicated that the ABM was sufficient for localizing proteins to the AIS. However, almost 10 years passed before Gasser et al. demonstrated that the ABM was in fact necessary and sufficient for Na_v_1.6 localization at the AIS in vivo and in vitro [78]. Mutation of a single Na_v_1.6 amino acid (E1100A) centered within the ABM disrupted the localization of the channel not only to AIS but also to Nodes of Ranvier (in DRG neurons) [78]. Interestingly, no significant changes in electrophysiological properties were detected with this point mutant even though AnkG binding increases persistent current [79] and the equivalent residue in Na_v_1.5, when mutated, induces Brugada syndrome [80]. In summary, several studies support the pivotal role of AnkG binding in the targeting of Na_v_ channels to the AIS and the maintenance of neuronal polarity. Interestingly, CK2, a kinase able to form complexes with Na_v_ channels, regulates AnkG-Na_v_ interaction via phosphorylation within the ABM. Inhibition of CK2, reduces the localization of both Na_v_1 and AnkG to the AIS [81,82].

**Figure 2.**
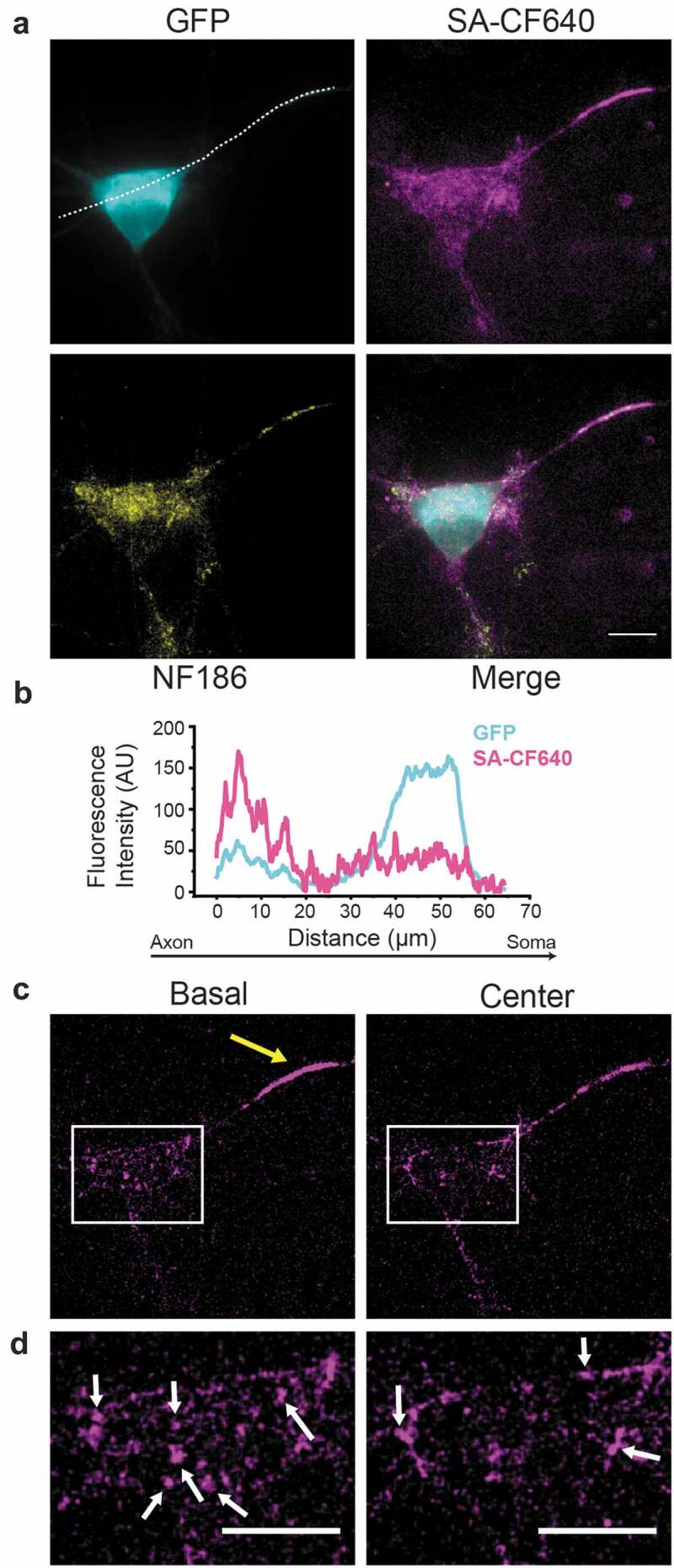
**Na_v_1.6 is concentrated at the AIS of rat hippocampal neurons (rHN), while presenting a punctate pattern (nanoclusters) on the soma**. Transfected DIV7 rHN expressing Na_v_1.6 tagged with an extracellular biotinylation site and cytoplasmic GFP. **A)** Average projections of a z-stack acquired with a spinning disk confocal of GFP (cyan), surface labeled Na_v_1.6 channels (magenta), and NF186 (yellow). White dashed line defines the profile presented in B. **B)** Line profile of the fluorescence intensities of GFP and surface labeled channels across the axon and somatic compartment. **C)** Individual images corresponding to basal (left) and center (right) planes of the same neuron shown in panel A. Large yellow arrow indicates the AIS. **D)** Enlargement of the somatic compartments (white squares shown in panel C). White arrows indicate somatic Na_v_1.6 nanoclusters. All scale bars represent 10 µm.

In addition to highly concentrating at the AIS, Na_v_1.6 also concentrates within nanodomains in the somato-dendritic compartment (Figure 2). Specifically, Na_v_1.6 forms nanoclusters of ≈230 nm on the soma of cultured rat hippocampal neurons expressing epitope-tagged Na_v_1.6. These structures are stable in place for over an hour but slowly gain and lose individual channels over this time frame [44]. While clustering is a prerequisite for coupled-gating, which has been suggested for some Na_v_ channels [83], the functional significance of these somatic nanoclusters is unknown at this time. However, this localization is known to be AnkG independent since a Na_v_1.6 ABM mutant that does not localize to the AIS still forms somatic nanoclusters [44]. This result indicates multiple mechanisms underlie Na_v_ channel localization in neurons and adds to the mystery of neuronal complexity. How do neurons maintain such different localizations of Na_v_1.6? Is it a matter of different interaction partners? Is the decision made at early stages of biosynthesis, at the vesicular transport level, or at the plasma membrane? How functionally different are somatic Na_v_1.6 channels as compared to those in the AIS or Nodes of Ranvier?

### Na_v_ trafficking mechanisms

Na_v_ channels, as most other transmembrane plasma membrane proteins, are translated in the rough endoplasmic reticulum (ER). If quality control misfolding checkpoints are passed, and after glycosylation (which takes place in both the ER and Golgi apparatus), they are trafficked to the neuronal surface via vesicular transport [84,85]. In fact, there are several disease-related mutations in Na_v_1.6 that are good examples of the importance of such early biosynthesis steps (see section below for more details).

**Figure 3.**
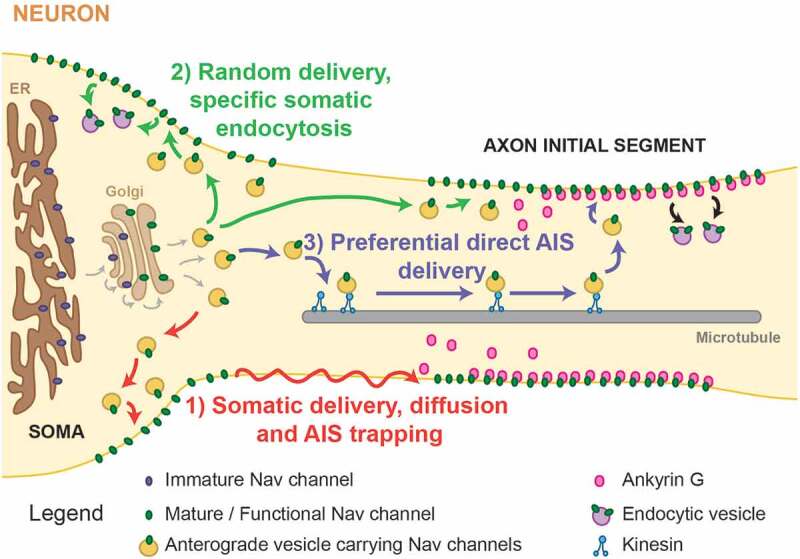
**AIS localization and Na_v_ trafficking mechanisms**. Possible trafficking mechanisms involved in Na_v_ localization in the somatic (left) and AIS (right) compartments: 1) preferential somatic delivery followed by lateral diffusion into the AIS and immediate immobilization upon AnkG binding (red arrows); 2) random delivery of protein into both compartments, followed by specific somatic endocytosis (green arrows); 3) directed delivery into each compartment with a preference for the AIS (blue arrows).

Specific mechanisms must exist in order to guarantee the delivery of a specific ion channel to a determined compartment such as the soma or the AIS. The transport of Na_v_ channels to the cell surface and how neurons accomplish and maintain such a polarized distribution has been under debate since the early 2000s. As summarized in Figure 3, at least three different mechanisms could target Na_v_ channels to the soma and AIS: 1) preferential somatic delivery followed by lateral diffusion into the AIS and immediate immobilization upon AnkG binding; 2) random delivery of protein into both compartments, followed by specific somatic endocytosis; and 3) directed delivery into each compartment with a preference for the AIS due to the requirement here for a high channel concentration. However, given the complexity of biology, it is likely some combination of all three mechanisms exists.

The AIS being a diffusion trap (trapping channels that were delivered initially in the somatic compartment [86]) (mechanism 1) avoids the need for any vesicular delivery into the axon in order to get Na_v_ channels into the AIS. However, this idea requires free diffusion between the soma and AIS. Experiments performed in the early 90s indicated that a plasma membrane diffusion barrier exists between the somatic and axonal compartments [87,88]. The physiological role of this barrier was naturally assumed to be central to neuronal polarization. This barrier blocked the trans-compartment diffusion of not only membrane proteins but also membrane lipids, and was fully established in cultured neurons by DIV10, as demonstrated by single particle tracking experiments performed in the early 2000s with labeled phospholipids and ion channel chimeras [89,90]. It is difficult to imagine how a large membrane protein such as a Na_v_ channel could make it across this barrier in a mature neuron. However, since Na_v_ channel expression begins well before DIV7 [89] this mechanism could play a role in the early development of the AIS.

The random delivery and soma specific endocytosis mechanism (mechanism 2) was supported by experiments performed with CD4-Na_v_1.2 chimeras in both COS7 cells and hippocampal neurons [91]. Garrido et al. showed that the C-terminal motif of Na_v_1.2 governed the axonal compartmentalization of a CD4-Na_v_1.2C-terminus chimera, which was inserted into both the somato-dendritic and the axonal compartments and was later removed from the soma but retained in the AIS. Within the Na_v_1.2 C-terminus resides a di-leucine motif (IL 1857–1858), which was proposed to be involved in clathrin-dependent endocytosis of the chimera from the somatic compartment and dendrites. The idea at the time was that either axonal Na_v_1.2 channels were prevented from endocytosis due to binding an axonal partner (such as AnkG) or that the axonal and somato-dendritic endosomal pathways were differentially regulated. Interestingly, although Na_v_1.1 and Na_v_1.6 also contain such di-leucine motif, it did not seem to play a role in these channels’ localization. The authors speculated that this could be due to either this motif not being exposed in the case of Na_v_1.1 or Na_v_1.6 (due to divergence in the C-terminal domain sequence and different conformations that may be adopted) or the different isoforms interacted with different endogenous partners. Also, related to Na_v_ channel endocytosis, Na_v_ channels contain a PY motif within their C-terminal domain which can be ubiquitinylated by Nedd4 and Nedd4-2, and which induce protein degradation [92]. Fotia et al. demonstrated that Na_v_1.2 and Na_v_1.7 were ubiquitinylated and, in *Xenopus* oocytes, while Nedd4-2 decreased the activity of Na_v_1.2, Na_v_1.7 and Na_v_1.8, Nedd4 only inhibited Na_v_1.2 and Na_v_1.7, not Na_v_1.8. Such endocytosis mechanisms were suggested to be involved in the Na_v_ channel polarized distribution in neurons. Some years later, Fache et al., continued supporting the idea of random insertion and somatic endocytosis with the publication that another motif (17 aa upstream of the ABM) was required for endocytosis of CD4-Na_v_ chimeras (in COS7 and neuronal cells) [93], and that probably the high concentration of AnkG in the AIS tethered the CD4-Na_v_1.2 II-III linker chimera and protected it from endocytosis. However, a concern with these results centered on whether the mechanism described for the CD4 chimeras would be translatable to the full-length Na_v_1.2. Indeed, this is why Gasser et al. performed the experiments with full Na_v_1.6 channel previously described. Although these investigators did not directly address delivery or endocytosis, they did demonstrate that AnkG binding was necessary and sufficient for native Na_v_1.6 localization at the AIS [78]. Importantly, they also paved the way for trafficking experiments with complete Na_v_ channels.

In order to address Na_v_ channel delivery directly into the AIS membrane (mechanism 3), our lab developed fluorescent protein tagged Na_v_1.6 channels that also carried a biotinylation site on the extracellular surface [56]. Biotinylation of this extracellular lysine occurred in the ER lumen during biosynthesis, thus these channels were biotinylated before they reached the plasma membrane. This extracellular biotin allowed for the specific detection of surface channels in living neurons using fluorescently conjugated streptavidins (see Figure 2 for an example). Importantly, if cultured neurons were incubated with fluorescent streptavidin during high signal to noise TIRF imaging, the delivery location of nascent channels to the cell surface would be detected with single molecule sensitivity [56]. Using this approach, we demonstrated that the full length Na_v_1.6 was preferentially inserted into the AIS membrane via direct vesicular trafficking (up to 6 channels/vesicle). Once inserted at the AIS plasma membrane, channels were immediately immobilized, as demonstrated by both FRAP and single particle tracking experiments. Such immediate immobilization did not occur to channels that were delivered at the somatic compartment. Furthermore, single particle tracking experiments also proved that no channels crossed the somato-axonal barrier, further arguing against the diffusion trap mechanism discussed above. An ABM Na_v_1.6 mutant was not preferentially delivered to the AIS, and the channels that were delivered to AIS were not immobilized. It was surprising that this mutant was delivered to the AIS at all since the Gu lab had demonstrated that AnkG links Na_v_1.2 to KIF5 to facilitate vesicular transport into the axon [94]. Thus, it appears different Na_v_ isoforms are trafficked via distinct mechanisms. Interestingly, although the ABM motif was required for preferential delivery to the AIS it did not confer preferential AIS delivery when added to a Na_v_1.2-K_v_2.1 chimera, indicating other channel domains are likely involved [56]. In addition, the use of the surface-specific label for Na_v_1.6 (biotinylated loopBAD tag, labeled with CF640-conjugated streptavidin), combined with the sensitivity of TIRF imaging, indicated that besides its concentration at the AIS, Na_v_1.6 presents two populations in the somatic compartment, a static nano-clustered pattern against a background of freely diffusing Na_v_1.6 molecules (see Figure 2). Nano-cluster formation was still observed with the ABM mutant incapable of binding AnkG [44,56]. In summary, our newly developed Na_v_1.6 constructs, which permitted the live imaging of single channels on the neuronal surface, allowed for both the mapping of membrane insertion at the AIS and the discovery of ankyrin-independent Na_v_1.6 localization on the somatic surface.

**Figure 4.**
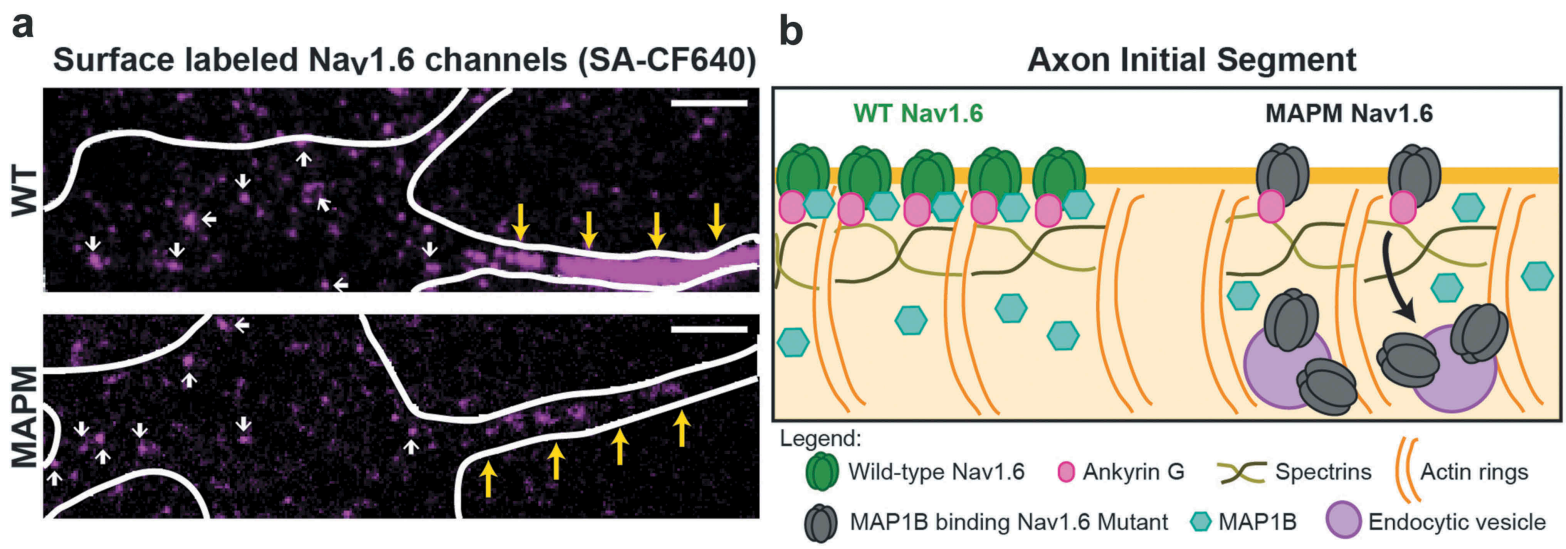
**MAP1B binding prevents Na_v_1.6 AIS compartment-specific endocytosis. A)** TIRF images of DIV10 rHNs transfected with WT (top) or MAPM (bottom) Na_v_1.6LoopBADGFP (only surface labeled channels with CF640-conjugated streptavidin (SA-CF640) are shown). White line depicts the cell outline. Yellow arrows point to the AIS (identified by NF186 labeling (data not shown)). Smaller white arrows point to somatic nano-clusters. Notice that while no significant differences are noticeable in the somatic labeling, there is a drastic decrease in the AIS labeling with MAPM versus WT channels. Scale bars represent 5 µm. **B)** Schematic drawing showing that while AIS WT Na_v_1.6 channels (green), are not endocytosed, AIS MAPM channels (p.VAVP(77–80)AAAA) (black) are, although they can still bind to AnkG.

The AIS delivery experiments just described suggested that endocytosis plays little role in the compartmentalization of Na_v_1.6 localization. However, biology is not always simple, as was reinforced when we applied our imaging technology to Na_v_1.6 mutants previously reported to have forward trafficking defects. One such Na_v_1.6 mutant (MAPM) carried amino acid substitutions in its MAP1B binding domain [28]. MAP1B light chain interaction with wild-type Na_v_1.6 was demonstrated by both yeast two-hybrid experiments and co-immunoprecipitation from mouse brain membranes. The MAP1B binding motif was defined in the N-terminal domain of Na_v_1.6 (VAVP, amino acids 77–80) [95]. It was initially postulated that MAP1B binding was necessary for delivery of Na_v_1.6 to the surface since removal of MAP1B binding prevented the detection of Na_v_1.6 currents in transfected ND7/23 cells. It was reasonable to assume that MAP1B binding was involved in the forward trafficking of Na_v_1.6 since, in addition to its classical role in microtubule stabilization, MAP1B is also known to regulate the localization, function and trafficking of different channels and receptors [96]. However, as our recent work demonstrated, and to our surprise, the Na_v_1.6 MAPM traffics to the somatic surface of hippocampal neurons but is not concentrated within the AIS (Figure 4(a)) [28]. While we expected that this distribution was due to a lack of trafficking to the AIS, the fluorescent biotinylation assay described above confirmed that this was not the case, with the MAPM being readily delivered to the AIS surface where it was immediately immobilized just like the wild-type channel. We found that MAPM is not concentrated at the AIS because, without MAP1B binding, it is internalized via compartment-specific Na_v_1.6 endocytosis [28]. Although MAPM channels are still tethered at the AIS plasma membrane by AnkG binding (as demonstrated by their restricted diffusion, similar to wild-type Na_v_1.6 channels), these channels are endocytosed from the axonal compartment when MAP1B is not able to bind to Na_v_1.6 (as illustrated in Figure 4(b)). Clearly, there is still much we do not understand about the regulation of the Na_v_-AnkG interaction. This trafficking work had an unexpected functional bonus as it also suggested that there is a correlation between Na_v_1.6 channel function and its location, where Na_v_1.6 persistent current is regulated by subcellular location. Both the MAPM and ABM mutants (both mainly somatic channels) presented five times more persistent current than wild-type Na_v_1.6, which accumulates at the AIS [28].

During the last two decades, the observation that somato-dendritic cargo-carrying vesicles do not enter the AIS has emphasized the importance/existence of a cytoplasmic filter regulating cargo entry into the axon [97–99]. This filter, which is formed during axon maturation (DIV3-5) [98], seems to be another complex mechanism formed by the coordination of different elements, one of them being the actin cytoskeleton, where dendritic protein-carrying vesicles are stopped [100,101]. This proximal pre-axonal exclusion zone (PAEZ) is also rich in MAP2 and TRIM46, is AnkG independent but dependent on microtubules and microtubule motors. Here, the combination of motor proteins associated with a particular vesicle determines what can enter the axon [102–105]. In addition, the recruitment of myosin-V to certain vesicles can induce vesicle immobilization at the PAEZ [106]. Hopefully, within the next years the detailed mechanisms underlying this selective cargo transport into the axon will be elucidated.

Another aspect of Na_v_ cell biology that needs more development centers on the molecular motors involved in vesicle transport. Although the role of kinesins in ion channel and AMPA receptor transport had been known since the mid 2000s [107,108], it wasn’t until 2013 that an interaction between KIF5 and a Na_v_ channel and forward trafficking was demonstrated [109]. Su et al. demonstrated that Na_v_1.8 interacts (directly or indirectly) with amino acids 511–620 in KIF5B, promoting surface channel expression via forward trafficking and also preventing channel degradation, without altering the biophysical properties of the channel [109]. Although KIF5A was also able to bind to Na_v_1.8, KIF5B showed a stronger affinity. Interestingly, KIF5 interacted with both Na_v_1.8 and Na_v_1.9 in dorsal root ganglia (DRG) neurons, but not with Na_v_1.6 or Na_v_1.7 [109]. A few years later, direct interaction between KIF5B and AnkG (but not AnkB) was reported, and AnkG was found to be required for Na_v_1.2 to be transported to the AIS. Na_v_1.2 co-transport with KIF5 was observed, as well as co-transport with AnkG in a subset of rat hippocampal neurons [94]. KIF5 and Na_v_1.2 bind distinct AnkG domains, which allows AnkG to act as an adaptor protein between the motor and channel. Unfortunately, in this study, no other Na_v_ channel isoforms were tested, so it is unknown whether Na_v_1.6 also needs to be bound to AnkG to be transported into the axon by KIF5, or whether it is transported by another kinesin. However, according to our experiments, an ABM Na_v_1.6 mutant was still delivered (although not preferentially) to the AIS [56], suggesting that this isoform does not require AnkG as an adaptor. Interestingly, a recent report indicates that Na_v_1.6 is transported to the surface of sensory neurons by a tripartite complex Na_v_1.6/δ-catenin/KIF3A (as observed by SIM super-resolution three color co-localization experiments) [110], although the authors did not determine whether such interactions were direct or indirect through other proteins. Palmitoylation of δ-catenin facilitated the membrane-directed trafficking of Na_v_1.6 in DRG neurons. They also showed by immunoprecipitation experiments that δ-catenin was able to bind to Na_v_1.6 and Na_v_1.7 (but not Na_v_1.8 nor Na_v_1.9) and KIF3A or KIF3B (but rarely KIF5A, KIF5B, KIF17 and KIFC2). Myosin-II (myh9 and myh10) has also been described to interact with Na_v_1.2, Na_v_1.3, Na_v_1.7 and Na_v_1.8, but not with Na_v_1.1, Na_v_1.6 nor Na_v_1.9 (according to immunoprecipitation experiments from rodent brain tissues) [111]. After analyzing the electrophysiological properties of Na_v_1.8 with and without myh10 in ND7/23 cells, where a 3-fold increase in current density and changes in activation and inactivation curves were observed, the authors suggested that non-muscle myosin-II could interact with Na_v_ channels to facilitate/regulate their transport and function.

### AIS plasticity

Besides its complex and organized structure, AIS location and composition is much less static than was initially appreciated. The AIS is dynamically regulated on both short and long term timescales, either by biophysical changes in ion channel function (which occur in the scale of milliseconds to seconds) or structural reorganization of the AIS (also called AIS plasticity, and which occurs in the scale of hours to days). These changes are expected to contribute to fine adjustments of cellular and network activity. Any mechanism involved in ion channel trafficking and AIS assembly is also likely to be involved in AIS plasticity.

The first report of AIS plasticity was by Grubb and Burrone in 2010. They reported that after depolarization (either treatment of hippocampal neurons with 15 mM KCl for 48 h, or photo stimulation-induced activation of channelrhodopsin2), the AIS moved up to 17 µm. This distal shift was also observed for AnkG, ßIV-spectrin, NF186, FGF14 and, most importantly, Na_v_ channels. AIS plasticity was Na_v_ activity independent (TTX did not prevent the shift) but dependent on Ca_v_ channels. AIS translocation was accompanied by decreased neuronal excitability and a correlation existed between the AIS start position and number of spikes generated, showing that neurons with a more proximal AIS presented a lower threshold for AP initiation and also more spikes in response to input current [112]. This publication was followed by another report showing that in avian brainstem auditory neurons, days after deprivation of auditory inputs, the AIS became longer, without affecting its proximal location and Na_v_ channel density [113]. This size increase lead to increased neuronal excitability. Interestingly, the extent of AIS elongation correlated with the levels of auditory/sensory attenuation that were induced. Such elongation was accompanied by changes in expression of K_v_ channels: K_v_7.2 increased while K_v_1.1 decreased, with no changes in K_v_3.1 [114]. Some years later, a detailed pharmacological screening performed by the Grubb laboratory concluded that AIS relocation in rat hippocampal cultures was triggered by activation of only L-type Ca_v_1 channels and CaMKII and was also calcineurin dependent (but calpain, PKA, PKC, MAPKK, p38 and PI3K independent) [115]. Interestingly, although calcineurin was necessary and sufficient for AIS relocation, the authors did not find a calcineurin concentration at the AIS.

Recently, the non-muscle myosin-II has been suggested to be necessary for AIS plasticity [116,117]. Treatment with blebbistatin (a myosin-II ATPase inhibitor) blocked both the activity-dependent translocation and shortening of the AIS induced by 15 mM KCl [116]. Interestingly, while myosin-II is present at the AIS, it is not concentrated there. However, the phosphorylated form of the myosin-II light chain (pMLC) is concentrated at the AIS, and in fact, pMLC is associated with the actin rings in the AIS [116,117]. Furthermore, as a result of a sustained depolarization, pMLC levels were specifically and rapidly decreased at the AIS, which preceded actin disorganization and loss of immuno-staining of AnkG and Na_v_ channels. The authors demonstrated that actin stabilization with either phalloidin or jasplakinolide prevented the activity-dependent loss of AnkG, but not the loss pMLC, suggesting that the loss of pMLC is regulated by a distinct mechanism [117].

Besides the slowly responding AIS plasticity just discussed (taking place within 3–6 hours and even days), fast AIS plasticity has also been reported. For example, high glutamate concentrations induced dynamin and clathrin-coated pit dependent endocytosis of K_v_7.2 and 7.3 and Na_v_ channels in hippocampal neurons within 10 minutes. These processes required the activation of NMDA receptors, resulting in calcium influx and calpain activation; while being calcineurin, PP1, PP2A and CaMKII kinase independent [118]. Such endocytosis seemed to be selective to AnkG-binding proteins, since AIS-localized GABA receptors were not endocytosed (although declustering was observed). It is important to note that the authors did not use a complete Na_v_ channel for this experiment, instead they used a SEP-TAC-Na_v_1.2 II-III loop chimera reporter (which bound to AnkG). Thus it is not clear whether their results would apply to full Na_v_1.2 and Na_v_1.6.

Forms of AIS plasticity have also been observed under pathological conditions. For example, AIS integrity is disrupted in ischemia (in a calpain-dependent manner) and during chronic microglial inflammation (a ROS-producing enzyme, NOX2-mediated and calpain-dependent process) [119–121]. Remodeling and shortening of the AIS length has been observed after stroke [122]. A shortening of the AIS in medial prefrontal cortex and hippocampus has also been observed in *db/db* mice (an established animal model for type-2 diabetes), which could lead to a decreased neuronal excitability that could explain cognitive and mood impairments associated with type-2 diabetes [123]. AIS plasticity has also been observed in cases of demyelination, epilepsy and Angelman syndrome (a neurodevelopmental disorder associated with autism) (for a review see [124]). AIS plasticity seems to be bidirectional and a homeostatic process that allows neurons to control their excitability and neuronal output [125]. It has also been theorized that since neuronal excitation increases during aging, aged neurons may present a shorter and/or more distal AIS. Although controversial, there are a couple of reports that support this theory [126,127].

AIS plasticity appears to be somehow cell-type-specific. For instance, all excitatory hippocampal neurons seem to present AIS plasticity, while GABAergic inhibitory hippocampal neurons do not [115]. When dopaminergic neurons from olfactory bulb (OB) were depolarized for 24h, their AIS lengthened and relocated proximally toward the soma, contrary to what was observed in other non-GABAergic OB neurons and hippocampal cells (shorter length and distal shift). Such translocation was dependent on L-type Ca_v_ channels but calcineurin, CaMKII and PKA independent. OB dopaminergic neurons treated with TTX for 24h did not present a shift but the AIS became shorter [128]. AIS plasticity has also been observed in human neurons. While chronic depolarization shortened the AIS of human cortical excitatory neurons [129], a distal shift of the AIS was observed in hiPSC-derived neurons (heterogeneous culture of excitatory and inhibitory neurons) [130]. Curiously, the shortening of human AIS was related to EB3 levels.

Furthermore, there is differential ion channel expression between distinct cell types, which also changes during development along with AIS location and structural components [131]. For example, while Na_v_1.2 is expressed early in the AIS during development, it is later displaced by Na_v_1.6, as the AIS becomes shorter and more distal. Furthermore, Na_v_1.2, like Na_v_1.1, presents a more proximal location than Na_v_1.6 even though AnkG is evenly distributed along the AIS [132,133]. Taken together these facts, and our recent discovery that MAP1B binding to N-terminal of Na_v_1.6 prevents its endocytosis from the AIS [28], raises the possibility that MAP1B could play a role in this differential isoform location or even AIS plasticity. Na_v_1.6 is the only isoform with a VAVP sequence in its MAP1B binding motif and yeast two-hybrid experiments showed that the Na_v_1.1 N-terminal domain (with a VSEP) does not bind MAP1B [95]. It will be interesting to see if the MAP1B endocytosis-shielding effect is extended to other isoforms and whether it plays a role in AIS plasticity (although bulk endocytosis does not seem to play a role in AIS shortening [116]).

### Impaired Na_v_ trafficking/AIS related disease-causing mutations

Most channelopathies (diseases caused by ion channel defects) are due to mutations affecting channel function, trafficking, or both. Most Na_v_ mutation-causing diseases are associated with a change in the conducting properties of the channel rather than altered trafficking (for reviews see [134–138]), but in this section we will focus on the latter. As mentioned earlier, disruption of AIS Na_v_ localization has been observed in cases of ischemia, demyelination, epilepsy and Angelman Syndrome [124,139].

Since early Na_v_ biosynthesis steps (exiting the ER and Golgi in order to reach to the plasma membrane) are crucial, it is not surprising that several disease-related mutations in Na_v_1.6 are caused by trafficking pathway defects. For example, the mouse *ataxia3*-causing mutation S21P in Na_v_1.6 does not generate sodium currents when expressed in ND7/23 cells unless cells are incubated at 30°C instead of 37°C, indicating a temperature-dependent trafficking defect. Immuno-staining of primary cerebellar granule cells from the *ataxia3* mice showed that the mutant was retained at the cis-Golgi level [140]. The human epilepsy-causing mutation R223G in Na_v_1.6 also leads to almost no channel expression and it is also partially rescued by incubating cells at 30°C instead of 37°C [141]. On the other hand, the spontaneous mouse mutation I1750Δ generates reduced sodium channel activity, due to defective glycosylation of the channel that results in improper localization at AIS and Nodes of Ranvier, leading to mice with movement disorders [142].

Na_v_1.6 is not the only Na_v_ channel where mutations that impair trafficking cause disease. For example, R1648C and G1674R Na_v_1.1 mutants (associated with Dravet Syndrome [143]) presented less surface channel and reduced current density when expressed in HEK293 cells. Interestingly, incubation with antiepileptic drugs for 24h increased surface expression in a mutant specific manner. For example, phenytoin and lamotrigine promoted trafficking of R1648C, while only phenytoin affected the G1674R mutant [143]. Furthermore, M1841T Na_v_1.1, a mutation associated with familial epilepsy, is a loss-of-function mutation that impairs forward trafficking to the surface, but surface expression can be restored by incubation at <30°C [144]. Mutation D1639N in Na_v_1.8, identified in a human patient suffering from a chronic pain syndrome (Small Fiber Neuropathy) [145], impairs trafficking of Na_v_1.8 to the plasma membrane [146]. Interestingly, the authors were able to restore its trafficking, in vitro, by overnight treatment with lidocaine. Mutations in Na_v_1.5 that affect either glycosylation or protein folding of the cardiac channel are associated with Brugada syndrome and Long QT syndrome [147–149]. In some cases, trafficking was improved by treatment with mexiletine.

Trafficking based diseases can be caused by mutations in proteins other than Na_v_ channels themselves. For example, AnkG polymorphisms are associated with a higher risk of bipolar disorder [150,151] and intellectual disabilities [152]. Recently, three human mutations in ANK3 (the gene encoding for the 480 KDa isoform of AnkG), caused improper AIS development manifested by a reduced Na_v_ channel and NF186 concentration within AIS [153]. These mutations are associated with neurodevelopmental disorder with delayed speech, intellectual impairment, and seizures, among other phenotypes.

## Future directions

Although remarkable progress has been made during the last decade regarding Na_v_ trafficking and AIS structure, there are still numerous questions that remain to be answered. What is the functional role of the somatic Na_v_1.6 nanoclusters and how are they maintained in the absence of AnkG binding? What determines whether a Na_v_1.6 channel reaches the plasma membrane at the somatic or axonal compartment? Are these mechanisms specific for each Na_v_ isoform or do all isoforms use the same motors and/or adaptor proteins? What is the specific molecular machinery involved in AIS plasticity? For example, does axonal endocytosis and the regulation of MAP1B binding play a role in AIS plasticity? Hopefully within the next years some of these questions will be answered. A better understanding of the complexity of Na_v_ localization and AIS plasticity will shed light on how this neuronal compartment regulates neuronal activity in health and disease.
